# Screening and Identification of DnaJ Interaction Proteins in *Streptococcus pneumoniae*

**DOI:** 10.1007/s00284-013-0424-4

**Published:** 2013-08-02

**Authors:** YingYing Cai, WenJuan Yan, WenChun Xu, YiBing Yin, YuJuan He, Hong Wang, XueMei Zhang

**Affiliations:** Key Laboratory of Diagnostic Medicine Designated by the Ministry of Education, Department of Laboratory Medicine, Chongqing Medical University, Chongqing, China

## Abstract

*Streptococcus pneumoniae* DnaJ is recognized as a virulence factor whose role in pneumococcal virulence remains unclear. Here, we attempted to reveal the contribution of DnaJ in pneumococcal virulence from the identification of its interacting proteins using co-immunoprecipitation method. *dnaJ* was cloned into plasmid pAE03 generating pAE03-*dnaJ*-*gfp* which was used to transform *S. pneumoniae* D39 strain. Then anti-GFP coated beads were used to capture GFP-coupled proteins from the bacterial lysate. The resulting protein mixtures were subjected to SDS-PAGE and those differential bands were determined by matrix-assisted laser desorption/ionization time of flight mass spectrometry. We finally obtained nine proteins such as DnaK, Gap, Eno, SpxB using this method. Furthermore, to confirm the interaction between DnaJ and these candidates, bacterial two-hybrid system was employed to reveal, for example, the interaction between DnaJ and DnaK, Eno, SpxB. Further protein expression experiments suggested that DnaJ prevented denaturation of Eno and SpxB at high temperature. These results help to understand the role of DnaJ in the pathogenesis of *S. pneumoniae*.

## Introduction

The heat shock protein (HSP) genes are highly conserved in all eukaryotes and prokaryotes [9, 27]. *Streptococcus pneumoniae* DnaJ is a member of conserved HSP40 family, which has been reported to function in physiological and various stress processes. Recent studies supported the idea that DnaJ is a virulence factor. In *Escherichia coli*, *dnaJ* deficient mutants did not grow as well as the wild type at temperatures above 30 °C [33]; Takaya et al. [35] reported that *Salmonella enterica* serovar Typhimurium DnaJ was essential for invasion of epithelial cells and survival within macrophages. DjlA, a membrane-anchored DnaJ-like protein, functioned as chaperone was closely associated with pathogenesis in *Legionella dumoffii* [28]. *djlA* mutants displayed a reduced growth rate and showed a striking loss of cytotoxic activity against *Ruditapes philippinarum* hemocytes in vitro in *Vibrio tapetis* [21]. Besides, we noticed that D39Δ*dnaJ* mutant grew slowly in C+Y medium, and was defected in colonization of nasal and lung. Moreover, compared with mice infected with wild type D39, mice infected with D39Δ*dnaJ* survived much longer in murine models [6]. Nevertheless, it’s still unclear how DnaJ is implicated in the pneumococcal virulence.

In *E. coli*, DnaJ is a co-chaperone, together with DnaK and GrpE, composed of DnaK/DnaJ system, which functions in folding and assembly of newly synthesized proteins, aggregation prevention, dissolution and refolding of aggregated proteins, translocation of proteins across membranes, and protein degradation [23]. Generally, DnaJ recognizes and binds substrates first, then stimulates ATP hydrolysis by Hsp70/DnaK. Also, DnaJ can recognize and bind substrate in the way independent of DnaK, such as the TorI RDF in *E. coli* [5].

Although previous studies have reported the substrate proteins interacting with DnaJ in some bacteria [13, 18, 36], this kind of proteins have not been reported in *S. pneumoniae*. Therefore, it’s very important to find DnaJ interaction proteins in *S. pneumoniae* which may facilitate the understanding the underlying mechanism of DnaJ implicated in pneumococcal virulence.

Here, we constructed a recombinant plasmid pAE03-*dnaJ*-*gfp*, which was used to capture the interaction proteins in *S. pneumoniae* by the method of co-immunoprecipitation. Using this method, we finally obtained nine proteins that would possibly interact with DnaJ in *S. pneumoniae* strain D39 by MALDI-TOF MS. The interactions between DnaJ and DnaK, Eno, SpxB were confirmed with bacterial two-hybrid system. Further protein expression experiments showed that DnaJ prevented denaturation of Eno and SpxB at high temperature and might also facilitate the secretion to extracellular matrix of Eno. The results provide evidence for the understanding the role of DnaJ in the pathogenesis of *S. pneumoniae*.

## Materials and Methods

### Bacterial Strains and Growth Conditions

*Streptococcus pneumoniae* strain D39 (NCTC 7466, serotype 2) was obtained from the National Collection of Type Cultures (NCTC, London, UK). Pneumococci were routinely grown in C+Y medium or on blood agar (BA) plates under microaerophilic conditions at 37 °C. Erythromycin (0.25 μg/ml) and chloromycetin (0.25 μg/ml) were added to the culture medium as needed.

### Construction of Recombinant Plasmids and Transformation into *S. pneumoniae* D39

Plasmid derivatives pAE03 were integrated to the *S. pneumoniae* chromosome at the native gene locus by single cross-over, which allowed the expression of C-terminal DnaJ-GFP+fusion protein.

The C-terminal part of target sequence was amplified by PCR from *S.* *pneumoniae* D39 with primers *dnaJ 1* and *dnaJ 2*, which incorporated the flanking restriction enzyme sequences of *Not* I and *Nhe* I (TaKaRa, China) restriction sites (Table 1). After digestion with *Not* I and *Nhe* I, the resulting fragment was then cloned into plasmid pAE03and transformed into *E. coli* BL21 (TaKaRa, China). The recombinant plasmid was transformed into *S. pneumoniae* D39 with 100 ng/ml synthetic CSP-1 peptide [29]. Positive strains (D39-*dnaJ*-*gfp*) were selected on BA plates supplemented with 0.25 μg/ml erythromycin, and confirmed by PCR with primers *gfp1* and *gfp2*. Primers *gfp1* and *gfp2* were designed from plasmid pAE03 itself. The full length of *eno* and *spxB* were amplified by PCR from *S.* *pneumoniae* D39 with primers *eno 1*, *eno 2* and *spxB 1*, *spxB* *2* separately, which incorporated the flanking restriction enzyme sequences of *Bgl* II and *Sma* I (TaKaRa, China) restriction sites (Table 1). After digestion with *Bgl* II and *Sma* I, the resulting fragment was then cloned into plasmid pEVP3 and transformed into *E. coli* BL21 (TaKaRa, China). The recombinant plasmid was transformed into *S. pneumoniae* D39 and D39Δ*dnaJ* mutant with 100 ng/ml synthetic CSP-1 peptide [29]. Positive strains (D39-pEVP3-*eno*/*spxB* or D39Δ*dnaJ*-pEVP3-*eno*/*spxB*) were selected on BA plates supplemented with 0.25 μg/ml chloromycetin, and confirmed by PCR with primers *chlo1* and *chlo2*. Primers *chlo1* and *chlo2* were designed from plasmid pEVP3 itself. All primers were listed in Table 1.

**Table 1:** Bacterial strains, plasmids and primers used in this study

Strains	Descriptions or sequences	Sources or references
*S. pneumoniae* strain D39	NCTC 7466, serotype 2	The National Collection of Type Cultures (NCTC, London, UK)
D39Δ*dnaJ* mutant	*dnaJ* deletion mutant of D39	This study
D39-*dnaJ*-*gfp*	D39 containing pAE03-*dnaJ*-*gfp*, that is *dnaJ*:: *gfp* fusion	This study
D39-pEVP3-*eno*	D39 containing pEVP3-*eno*, that is pEVP3:: *eno* fusion	This study
D39-pEVP3-*spxB*	D39 containing pEVP3-*spxB*, that is pEVP3:: *spxB* fusion	This study
D39Δ*dnaJ*-pEVP3-*eno*	D39Δ*dnaJ* containing pEVP3-*eno*, that is pEVP3:: *eno* fusion	This study
D39Δ*dnaJ*-pEVP3-*spxB*	D39Δ*dnaJ* containing pEVP3-*spxB*, that is pEVP3:: *spxB* fusion	This study
*E. coli* BL21	Carry recombinant plasmids	TaKaRa, China
Plasmids		
Plasmid pAE03	Erm^r^	Jan-Willem Veening
Plasmid pEVP3	Cam^r^	M. Donald
Primers(from 5′ to 3′)		
Primer sets for co-IP		
*dnaJ* 1	ATAAGAATGCGGCCGCAGACAAGTTTGAACGTGAAGGAAC GA	This study
*dnaJ* 2	CTAGCTAGCTTCTCCATCAAAGGCATCTT TAATA	This study
*gfp*1	AAAGGAGAAGAACTTTTCACTGGAG	This study
*gfp*2	AGTAGTGACAAGTGTTGGCCATGGA	This study
Primers for β-galactosidase reporter gene assay		
*eno* 1	GAAGATCTATGTCAATTATTACTGATGTTTACG	This study
*eno* 2	TCCCCCGGGTTTTTTAAGGTTGTAGAATGATTTC	This study
*spxB*1	GAAGATCTATGACTCAAGGGAAAATTACTGCAT	This study
*spxB*2	TCCCCCGGGTTTAATTGCGCGTGATTGCAATCCT	This study
*chlo1*	TTATAAAAGCCAGTCATTAGGCCTA	This study
*chlo2*	ATGAACTTTAATAAAATTGATTTAG	This study
Primers for bacterial two-hybrid system		
pBT-*dnaJ* 1	ATAAGAATGCGGCCGCAATGAACAATACTGAATTT	This study
pBT-*dnaJ* 2	CCGCTCGAGTTATTCTCCATCAAAGG	This study
pTRG-*dnaK* 1	ATAAGAATGCGGCCGCAATGTCTAAAATTATCGGTATTGACT	This study
pTRG-*dnaK* 2	CCGCTCGAGTTACTTTTCCGTAAACTCTCCGTCT	This study
pTRG-*eno* 1	CGCGGATCCATGTCAATTATTACTGATGTTTACG	This study
pTRG-*eno* 2	CCGCTCGAGTTATTTTTTAAGGTTGTAGAATGAT	This study
pTRG-*spxB* 1	CCGGAATTCAGATGACTCAAGGGAAAATTACTGCAT	This study
pTRG-*spxB* 2	CCGCTCGAGTTATTTAATTGCGCGTGATTGCAAT	This study

### Western Blot Analysis

*Streptococcus pneumoniae* strain D39 and D39-*dnaJ*-*gfp* were cultured in C+Y medium until an OD_600_ = 0.4–0.5. Bacteria were collected by centrifugation at 8,000 rpm for 10 min and washed twice with phosphate-buffered saline (PBS, pH 7.4). The pellet was then resuspended in 4 ml PBS, followed by adding appropriate protease inhibitor cocktail (BBI) to inhibit protein degradation. Then, the pellet was sonicated for 15–20 min and cell debris was removed by centrifugation at 12,000 rpm for 30 min. The supernatant was collected and used for further studies. *S. pneumoniae* strain D39 and D39Δ*dnaJ* were cultured in C+Y medium until an OD_600_ = 0.4–0.5. Erythromycin (0.25 μg/ml) was added to the latter culture medium. Bacteria were collected by centrifugation at 12, 000 rpm for 2 min and washed twice with PBS. The pellet was then resuspended in 200 μl 2× SDS loading buffer, followed by boiling 10 min and the supernatants were collected by centrifugation at 13,000 rpm for 2 min.

The proteins were electroblotted onto polyvinylidene difluoride (PVDF) membranes, blocked with 5 % skim milk (Sigma), and then probed with a 1:300–500 dilution of GFP antibody (Beyotime), 1:8,000 dilution of anti-DnaJ antiserum, 1:400 dilution of anti-CodY antiserum, 1:1,000 dilution of anti-Eno antiserum or 1:2,000 dilution of anti-SpxB antiserum. Polyclonal anti-DnaJ and anti-CodY/Eno/SpxB antiserum were raised in rabbit and mouse by routine immunogenic procedures separately [11, 38]. The secondary antibody was a 1:5,000 dilution of goat anti-rabbit or mouse immunoglobulin G conjugated to horseradish peroxidase (HRP, Promega). Chemiluminescence was used to detect HRP-conjugated secondary antibody used in western blots.

### Co-Immunoprecipitation (co-IP)

The procedures were performed essentially described as the protocol for GFP antibody (A.v. Monoclonal Antibody, Clontech) with some modifications [4, 12, 32]. Briefly, 1 ml cleared lysate of D39 and D39-*dnaJ*-*gfp* were transferred to a 10-ml beaker separately. 500 μl protein G-agarose beads (GE) which were prewashed with PBS twice were added to the beaker and incubated together for 3 h at 4 °C on a rotating apparatus with magnetic stirrer to remove non-specifically bound proteins. The beads were spined down and the supernatants were transferred to another 10-ml beaker. 20–25 μg GFP antibody (Clontech) was added to the supernatants and incubated at 4 °C for 1 h beforehand. Then 800 μl protein G-agarose beads were added to the supernatants and incubated overnight at 4 °C on a rotating apparatus. The beads were spined down and the supernatants were removed. The beads were washed five times with 1 ml PBS for 2 min each wash. The supernatant was discarded from final wash and the pellet was resuspended in 50–80 μl 2× SDS sample buffer. The samples were boiled for 5 min and spined down. 10–15 μl of the supernatant was loaded on an SDS/polyacrylamide gel and continued with western blot by GFP antibody detection.

### Matrix-Assisted Laser Desorption/Ionization Time of Flight Mass Spectrometry (MALDI-TOF MS) analysis

Samples from co-IP were analyzed by SDS-PAGE, and then stained with Coomassie brilliant blue (CBB G-250). Differential protein bands between D39 and D39-*dnaJ*-*gfp* were sent to BGI Tech Solutions Co., Ltd (Shenzhen, China) for MALDI-TOF MS analysis.

### Bacterial Two-Hybrid System

A bacterial two-hybrid system utilizing bacteriophage λ repressor protein and RNA polymerase was used as previously described [7, 8, 15, 32]. The DnaJ was fused to the full-length bacteriophage λ repressor protein (λcI, 237 amino acids), containing the amino-terminal DNA-binding domain and the carboxylterminal dimerization domain, while DnaK or Eno, SpxB was fused to the N-terminal domain of the α-subunit of RNA polymerase (248 amino acids). pBT-*dnaJ* and pTRG-*dnaK/spxB/eno* were co-transformed into an XL1-Blue MRF’ Kan strain. Cells were plated on nonselective, selective, and dual selective screening plates all containing chloromycin (25 μg/ml) and tetracycline (30 μg/ml) and incubated at 37 °C. Activity of a reporter gene was monitored, as previously described [7, 8, 15, 32].

### β-Galactosidase Reporter Gene Assay

*Streptococcus pneumoniae* strain D39 and D39-pEVP3-*eno*/*spxB* or D39 Δ*dnaJ*-pEVP3-*eno*/*spxB* were cultured in C+Y medium until an OD_600_ = 0.4–0.5. Then 10^7^ CFU bacteria were collected by centrifugation at 12,000 rpm for 2 min, the supernatant was collected as needed, and washed twice with PBS. The pellet was then resuspended in 500 μl 0.1 % triton X-100 dissolved in PBS, followed by reacting 15 min at room temperature and 50 μl reaction product was taken for detection following the instructions of β-galactosidase reporter gene assay kit (Beyotime, China), finally the protein expressions were detected in Microplate reader at 450 nm.

### Statistical Analysis

Statistical differences between groups were analyzed by either the Student’s *t* test or two-way ANOVA depending on the data. A probability level less than 0.05 was considered significant, *P* < 0.05 was indicated by *; *P* < 0.01 was indicated by **; *P* < 0.001 was indicated by ***.

## Results

### Recombinant Plasmid pAE03-*dnaJ*-*gfp* Is Successfully Constructed and Transformed into D39

**Fig. 1:**
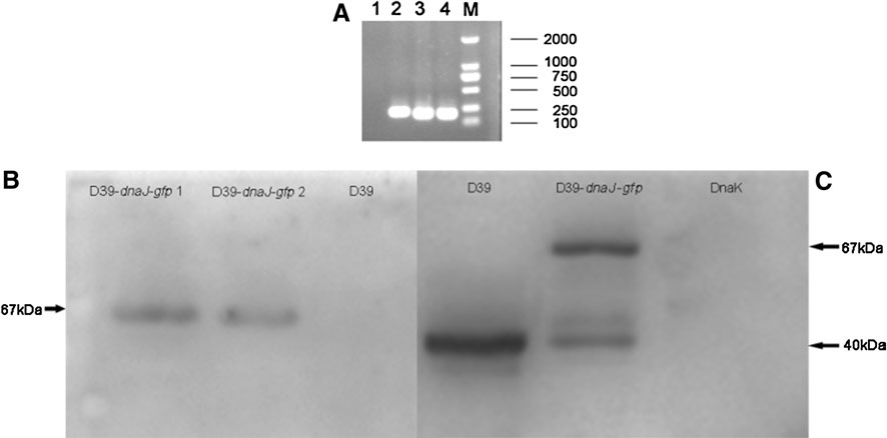
Identification of pAE03-*dnaJ*-*gfp* in *S. pneumoniae* strain D39-*dnaJ*-*gfp*. Two positive colonies (D39-*dnaJ*-*gfp*) were selected on BA plates supplemented with 0.25 μg/ml erythromycin, and confirmed by PCR with *gfp* primers (**a**). The samples were D39 (*1*), plasmid pAE03 (*2*), D39-*dnaJ*-*gfp* 2 (*3*), D39-*dnaJ*-*gfp* 1 (*4*) and marker (*M*).Then they were cultured in C+Y medium until OD_600_ 0.4–0.5. Bacteria was collected by centrifugation at 12,000 rpm and washed twice with PBS. The pellet was then resuspended in 50–80 μl 2× SDS sample buffer. Samples were boiled for 30 min and spined down. 10–15 μl of supernatant was loaded on an SDS-PAGE gel and continued with western blot probed by GFP antibody (Beyotime) (**b**) and anti-DnaJ antiserum (**c**) detection. DnaK protein was purified from *E. coli* BL21 (TaKaRa, China)

PCR and sequencing were used to confirm the successful construction of pAE03-*dnaJ*-*gfp* (Fig. 1a). The resulting plasmid was transformed into pneumococcal D39 strain, GFP or DnaJ-GFP fusion protein was detected with western blot probed with a 1:300–500 dilution of GFP antibody (Fig. 1b) or a 1:8,000 dilution of anti-DnaJ antiserum (Fig. 1c). When detected with GFP antibody, there was one band with a molecular weight (Mw) ~67 kDa in *S. pneumoniae* strain D39-*dnaJ*-*gfp*; no band could be detected in WT D39 strain (Fig. 1b). The ~67 kDa protein is coincident with the Mw of DnaJ-GFP fusion protein, because DnaJ has the Mw of approximate 40 kDa and the Mw of GFP is approximate 27 kDa. We observed two bands in D39-*dnaJ*-*gfp*, but only one band in D39, no band in negative control pure protein DnaK (Fig. 1c) probed with anti-DnaJ antiserum. The lower band indicated DnaJ, and the higher represented fusion protein DnaJ-GFP. Together, these results showed that plasmid pAE03-*dnaJ*-*gfp* has been successfully constructed and transformed into D39 which can be used for further studies.

### Screening and Identification of DnaJ Interaction Proteins in *S. pneumoniae* D39 by co-IP and MALDI-TOF

**Fig. 2:**
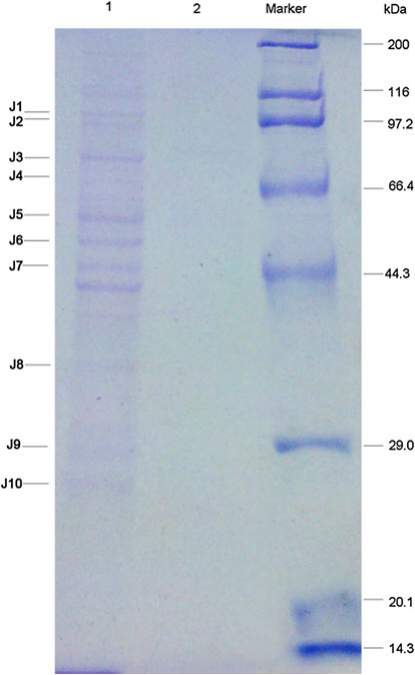
Differential DnaJ interaction proteins between *S. pneumoniae* strain D39 and D39-*dnaJ*-*gfp* by co-IP. *S. pneumoniae* strain D39 and D39-*dnaJ*-*gfp* were cultured in C+Y medium until OD_600_ 0.4–0.5. Then the bacteria were collected and the pellet was sonicated. Cell debris was removed by centrifugation at 12,000 rpm for 30 min. The supernatant was collected and used for co-IP. *Lanes 1* and *2* showed the proteins attached to protein G-agarose beads in *S. pneumoniae* D39-*dnaJ*-*gfp* or D39, respectively

The procedures of co-IP were mainly described as the protocol for GFP antibody with some modifications. As a result, we obtained 15 differential proteins attached on protein G-agarose beads between *S. pneumoniae* D39-*dnaJ*-*gfp* and WT D39 (Fig. 2).

To identify the differential DnaJ interaction proteins between D39 and D39-*dnaJ*-*gfp* and make sure the bands are reproductively distinguished from D39, we repeated co-IP experiment again and obtained 10 differential protein bands. The 10 bands were subjected to MALDI-TOF MS analysis. Those proteins included bi-functional acetaldehyde-CoA/alcohol dehydrogenase, *fusA* gene product/translation elongation factor G, chaperone protein DnaK, *spxB* gene product/pyruvate oxidase, *tuf* gene product/translation elongation factor Tu, *eno* gene product/phosphopyruvate hydratase, *gap* gene product/glyceraldehyde-3-phosphate dehydrogenase, type I, *rplC* gene product/ribosomal protein L3, and *rplF* gene product/ribosomal protein L6. Except for ribosomal proteins, the functions of seven proteins were basically defined, in particular the chaperone protein DnaK, which had been proved in many other bacteria as a DnaJ interaction partner [5, 21, 23, 28, 33, 35].

### DnaJ Interacts with DnaK, Eno, and SpxB in XL1-Blue MRF’ Kan Strain

Previous studies demonstrated the interaction between DnaJ and DnaK, especially in *E. coli* [10]. Combined with the co-IP results, it’s reasonable that DnaJ may also interact with DnaK and Eno, SpxB in *S. pneumoniae*. To test this hypothesis, we checked the interaction between them in XL1-Blue MRF’ Kan strain using the bacterial two-hybrid system [7, 8, 15, 32]. DnaJ was fused to the full-length bacteriophage λ repressor protein, pBT; whereas, DnaK, Eno, and SpxB were fused to the N-terminal domain of the α-subunit of RNA polymerase, pTRG separately. DnaJ was tethered to the λ operator sequence upstream of the reporter promoter through the DNA-binding domain of λcI. If DnaJ interacts with the candidate protein, they recruit and stabilize the binding of RNA polymerase at the promoter and activate the transcription of the *HIS3* reporter gene. *aadA* is a second reporter encoding a protein that conferred resistance against streptomycin to strengthen the evidence for the interaction between the bait and the target. pBT-*dnaJ* and pTRG-*dnaK/eno*/*spxB* were co-transformed into an XL1-Blue MRF’ Kan strain. Expression of fusion proteins was confirmed by western blot analysis. In control experiments, when the pBT and pTRG fragments alone were co-expressed, no colonies appeared on selective screening plates containing 5 mM 3-AT. When *dnaJ* and *dnaK/eno*/*spxB* were fused to both the pBT and pTRG fragments and co-expressed, colonies appeared on selective screening plates and dual selective screening plates containing 5 mM 3-AT and 12.5 μg/ml streptomycin.

**Fig. 3:**
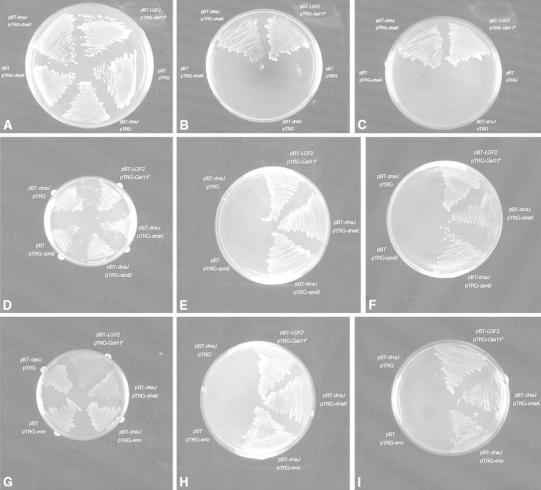
DnaJ interacts with DnaK, SpxB, and Eno in XL1-Blue MRF’ Kan strain in vivo. A bacterial two-hybrid system was used to confirm the interaction between DnaJ and DnaK/SpxB/Eno in vivo. Interaction between DnaJ and DnaK/SpxB/Eno was monitored by the expression of *HIS3* and *aadA* reporter genes on nonselective (**a**, **d**, **g**), selective (**b**, **e**, **h**) and dual selective (**c**, **f**, **i**) screening plates

Bacteria co-expressing DnaJ and DnaK/Eno/SpxB were viable on nonselective, selective, and dual selective screening plates as the positive control (Fig. 3a–i). In sharp contrast, there were no colonies on selective and dual selective screening plates when the pBT and pTRG-*dnaK/eno/spxB*, pBT-*dnaJ* and pTRG, or pBT and pTRG fragments were co-transformed respectively (Fig. 3b, c, e, f, h, i), although they could be seen on nonselective screening plates (Fig. 3a, d, g), indicating the fitness of the bacterial two-hybrid system. These results demonstrated the interaction between DnaJ and DnaK also works in *S. pneumoniae* and Eno/SpxB interacts with DnaJ in *S. pneumoniae*.

### Prevention of Denaturation of SpxB and Eno by DnaJ at High Temperature

**Fig. 4:**
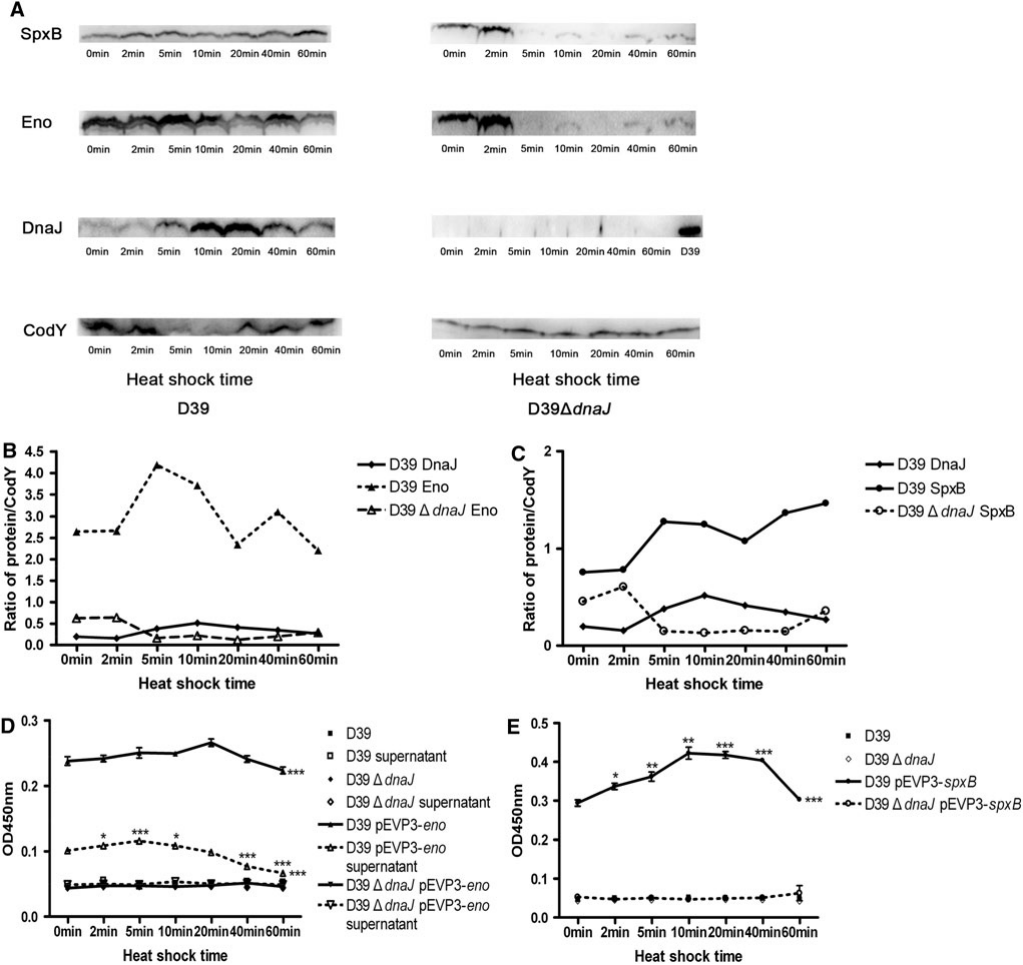
DnaJ prevents the denaturation of Eno and SpxB during heat shock. *S. pneumoniae* D39, D39Δ*dnaJ*, D39-pEVP3-*eno*/*spxB*, and D39Δ*dnaJ*-pEVP3-*eno*/*spxB* were cultured in C+Y medium and all the bacteria incubated in 42 °C water bath for 0, 2, 5, 10, 20, 40, and 60 min separately. DnaJ/SpxB/Eno expressions in the bacterial lysates of D39 and D39Δ*dnaJ* were analyzed by western blot separately. CodY served as the internal reference (**a**). Figure **b** and **c** were the ratios of DnaJ/Eno versus CodY and DnaJ/SpxB versus CodY. The results of β-galactosidase reporter gene assay were indicated by Fig. **d**, **e**, and the figures represented three independent experiments. Statistical differences were analyzed by two-way ANOVA between groups of D39-pEVP3-*eno*/*spxB* and D39, D39Δ*dnaJ* or D39Δ*dnaJ*-pEVP3-*eno*/*spxB*, respectively. Their *P* values were marked with *** located on the *right* of the *spots*. For D39-pEVP3-*eno*/*spxB* and D39-pEVP3-*eno* supernatant, statistical differences were compared by Student’s *t* test between samples at different time of heat shock and the initial time. Their *P* values were indicated above the *spots*. * indicates *P* < 0.05; ** indicates *P* < 0.01, and *** indicates *P* < 0.001

DnaJ is helpful to the correct functions of protein, we supposed whether the interacting partners were regulated by DnaJ in this way. Because the correct function of protein is necessary for the activity of β-galactosidase, β-galactosidase reporter assay was used to reflect the portion of functional proteins in the bacterial lysates and the supernatants, while western blot was employed to determine the total synthesized proteins in different bacterial lysates. We have known that DnaJ can facilitate some secretory proteins transport across the membrane, such as alkaline phosphatase (AP), ribose-binding protein (RBP), and β-lactamase (Bla) and metallo-β-lactamase (MβL) in *E. coli* [26, 36, 37]. And it has been reported that enolase can be secreted outside the bacteria [2], so we were also interested in the level of Eno in the supernatant. The amount of functional Eno was much more in wild type D39 strain than D39Δ*dnaJ* as revealed by β-galactosidase activity analysis (Fig. 4d), and the total synthesized Eno was also elevated with the increasing expression of DnaJ (Fig. 4a, b). Besides, we observed that the secretion of Eno was in parallel with the expression of DnaJ (Fig. 4b, d). It was suggested that besides its effect on the prevention of denaturation of Eno, DnaJ might also help to promote its outside secretion.

As to SpxB, the portion of functional SpxB was more in the amount in wild type D39 strain than D39Δ*dnaJ* (Fig. 4e), and decreased with the decline of DnaJ after heat shocked at 42 °C for 40 min (Fig. 4c, e), indicating that DnaJ was necessary to prevent the denaturation of SpxB despite the total synthesized SpxB was still increasing (Fig. 4c).

For D39Δ*dnaJ*, there were few SpxB and Eno expressions after heat shock for 5 min (Fig. 4a); while no functional proteins could be found through β-galactosidase reporter gene assays (Fig. 4d, e), indicating DnaJ might be important to protect SpxB and Eno from degradation.

## Discussion

*Streptococcus pneumoniae* encounters heat stress upon penetration from the nasal mucosa into blood and/or meninges. Commonly, infection with *S. pneumoniae* leads to elevated temperature in host, which serves as a key trigger for the rapid, transient increase in the synthesis of HSPs instead [20]. Therefore, HSPs are essential for the pathogenesis of pneumococcal infections. As a HSP member, DnaJ is believed to be associated with this heat shock event and confers fitness for bacterial survival. Definitely, DnaJ is associated with bacterial virulence, including *S. pneumoniae* [6, 21, 28, 35]. To investigate the underlying mechanism, we attempted to screen DnaJ interaction proteins with the method of co-IP. Finally, we obtained nine proteins with MALDI-TOF MS analysis.

Using co-IP to screen the interactive proteins is reliable. In the present study, we noticed the interaction between DnaJ and DnaK/Eno/SpxB using co-IP, which were confirmed by using bacterial two-hybrid reporter system. This is the first time that we report the interaction between DnaK and DnaJ in *S. pneumoniae*, albeit their interaction in other bacteria.

Our results showed several ribosomal proteins could also interact with DnaJ, indicating the diverse roles for DnaJ in protein synthesis in pneumococci. Therefore, DnaJ may be an important virulence factor which confers fitness for pneumococci to adapt host stress by interacting with the ribosomal proteins. This result is supported by the observation on other bacteria, such as *E. coli* and *Saccharomyces cerevisiae* [19, 24, 39]. Thus, DnaJ-mediated host adaptation may be a common phenomenon among bacteria.

The elongation steps are the most highly conserved processes for living bacterial cells, which was learnt from the comparisons of the processes among the initiation, elongation, and termination stages of protein synthesis [14]. In bacteria, the elongation steps of protein synthesis require the sequential action of two different elongation factors (EF), EF-G and EF-Tu. EF-Tu is required for delivering the correct aminoacyl-tRNA to the A site on the ribosome and is, therefore, intimately involved in proofreading [31]. Translation elongation factor EF-G uses GTP to catalyze translocation of peptidyl-tRNA from the ribosomal A/P site to the P/P site [25, 30]. After GTP hydrolysis and translocation, EF-G·GDP leaves the ribosome and is regenerated by the spontaneous exchange of GDP for GTP off the ribosome [17, 30]. EF-G·GTP also plays a role with ribosome recycling factor in splitting the ribosome into its two subunits after translation termination [16]. And, EF-G in the virulence has also been reported in *S. enterica* serovar Typhimurium [22]. Together these evidences suggest the possible role of DnaJ in bacterial virulence, which may be partly attributed to its ability in interaction with EF-G and Tu.

In pneumococcus, enzyme pyruvate oxidase (SpxB) is responsible for the production of H_2_O_2_ under rich and aerobic conditions. *S. pneumoniae* SpxB was in relationship with the bacterial transformation. The contribution of SpxB in virulence has also been reported. Spellerberg B et al. [34] demonstrated that a *spxB*-deficient mutant exhibited reduced virulence for nasopharyngeal colonization, pneumonia and sepsis. D39Δ*dnaJ* mutant was defected in colonization of nasal and invasive infections [6].Our results suggested that DnaJ prevented the denaturation of SpxB during heat shock, so in D39Δ*dnaJ* mutant SpxB may cannot express correctly and contribute to the reduction of colonization.

*eno* is an essential gene in bacteria and enolase, *eno* designated, is the key enzyme in the glycolytic cycle [2]. Besides, enolase can be secreted outside the bacteria and bind to plasminogen and plasmin to activate fibrinolysis system facilitating pathogen invasion and dissemination in the infected host [2]. Moreover, it also binds to human complement inhibitor C4b-binding protein and contributes to complement evasion [1]. Pneumococcal enolase mutant also exhibits attenuation in a model of respiratory infection [2]. Besides, the mouse intranasal challenge studies indicate that defined amino acid substitutions of Eno affect the virulence of *S. pneumoniae* and contribute to the pathogenesis of diseases [3]. In our study, we found the secretion of Eno changed with DnaJ and the total expression of Eno increased during 42 °C heat shock. Therefore, in D39 DnaJ may help enolase translocate to the extracellular matrix leading to invasive infection. Together, the implications of DnaJ in virulence appear to correlate with its effects in controlling the correct expression or effective secretion of some known virulence factors.

In conclusion, we obtained several proteins that could interact with DnaJ to execute normal physiological or biological functions. Of them, SpxB, Eno, and some translation related factors could be used to interpret the contribution of DnaJ in pneumococcal virulence. Nevertheless, the precise mechanism is worth further investigation.
